# Ultra-processed food consumption and increased risk of metabolic syndrome: a systematic review and meta-analysis of observational studies

**DOI:** 10.3389/fnut.2023.1211797

**Published:** 2023-06-09

**Authors:** Long Shu, Xiaoyan Zhang, Jianying Zhou, Qin Zhu, Caijuan Si

**Affiliations:** ^1^Department of Nutrition, Zhejiang Hospital, Xihu District, Hangzhou, Zhejiang, China; ^2^Department of Digestion, Zhejiang Hospital, Xihu District, Hangzhou, Zhejiang, China

**Keywords:** food processing, ultra-processed food, metabolic syndrome, meta-analysis, systematic review, observational study

## Abstract

**Background:**

Although higher consumption of ultra-processed food (UPF) has been linked to a higher risk of metabolic syndrome (MetS), the results remain controversial. Herein, we performed a systematic review and meta-analysis of observational studies to clarify the relationship between UPF consumption defined by the NOVA framework and risk of MetS.

**Methods:**

An extensive literature search on PubMed, ISI Web of Science, EBSCO and China National Knowledge Infrastructure (CNKI) databases was conducted to search for the relevant articles published priori to January 2023, and newly published articles between January 2023 and March 2023 were re-searched. Random-effects or fixed-effects models were adopted to calculate the pooled relative risks (RRs) and 95% confidence intervals (CIs). The between-study heterogeneity was explored using the Cochran's Q test and I-square (I^2^). Publication bias was investigated using the visual inspection of asymmetry in funnel plots and Begg's and Egger's tests.

**Results:**

Nine studies (six cross-sectional and three prospective cohort studies) totaling 23,500 participants with 6,192 MetS cases were included in the final analysis. The pooled effect size for the highest vs. lowest categories of UPF consumption indicated a positive association with the risk of MetS (RR: 1.25, 95%CI: 1.09–1.42, *P* < 0.0001). Subgroup analyses revealed a positive association between consumption of UPF and MetS risk in cross-sectional studies (RR: 1.47, 95%CI: 1.16–1.87, *P* = 0.002), and no significant association in cohort studies (RR: 1.10, 95%CI: 0.96–1.27, *P* = 0.104), respectively. In addition, a more significant association between UPF consumption and increased risk of MetS was found in the subgroups of study quality <7 (RR: 2.22; 95%CI: 1.28–3.84, *P* = 0.004) than study quality ≥7 (RR: 1.20; 95%CI: 1.06–1.36, *P* = 0.005). Similarly, when we performed analyses separately by sample size, there was a significant association between UPF consumption and MetS risk in sample size ≥5,000 (RR: 1.19; 95%CI: 1.11–1.27, *P* < 0.0001), and in sample size <5,000 (RR: 1.43; 95%CI: 1.08–1.90, *P* = 0.013), respectively.

**Conclusions:**

Our findings suggest that higher consumption of UPF is significantly associated with an increased risk of MetS. Further longitudinal studies are needed to confirm the effect of UPF consumption on MetS.

## Introduction

Metabolic syndrome (MetS) is a pathophysiological state and cluster of ≥3 different cardiovascular risk factors, including abdominal obesity, insulin resistance, elevated blood pressure and dyslipidemia (1). Parallel to rapid economic development and changes in lifestyle, the prevalence of MetS is continuing to rise worldwide, affecting approximately a quarter of the adult population, and has become an important public health issue (2, 3). The Chinese National Nutrition and Health Surveillance (2010–2012) reported that the prevalence of MetS in Chinese adults aged 20 years or older was 18.7% and an estimated 189 million adults living with MetS in China (4). Likewise, the prevalence of MetS in U.S. adults reached 34.2% during 2007–2012, according to the National Health and Nutrition Examination Survey (NHNES) (5). Meanwhile, MetS has also been considered to be an important risk factor for many chronic non-communicable diseases (NCDs), such as type 2 diabetes, stroke, cardiovascular disease (6). Given the high morbidity and its strong link to some NCDs, early prevention of MetS is of almost importance. Although the precise etiology of MetS is not completely understood, known risk factors for MetS included genetic predisposition, smoking, alcohol consumption, sedentary lifestyle and high sugar or fat diets (6, 7).

Over the past several decades, abundant evidence has shown that dietary factors play the important role in the development of MetS (8). Previous studies have specially examined the associations between the consumption of specific foods or nutrients and risk of MetS (9–11). However, less attention has been paid for the association between different degree of food processing and MetS. Recently, the global consumption of ultra-processed foods (UPF) has been rising rapidly in some middle- or high-income countries, contributing to 25%~60% of daily energy intake (12–14). During the SARS-CoV-2 pandemic, many people were at risk of financial restrictions, which could easily translate into choosing UPFs, which are typically high in energy density, added sugars, salt, saturated and trans-fats, as well as low in dietary fiber, protein, vitamins and minerals (15). Apart of nutritional composition, UPFs are highly palatable, convenient, long shelf life and affordable (14). Thus, UPF intake has garnered considerable attention from scientific researchers. To date, some observational studies have explored the potential associations between UPF consumption and various adverse health outcomes, including obesity, type 2 diabetes, hypertension, and cardiovascular disease (16–19). Of note, several recent systematic review and meta-analyses have also been published to clarify the associations between consumption of UPF and type 2 diabetes, hypertension and all-cause mortality (20–22). Consequently, these studies provided fairly consistent support for the positive associations of UPF consumption with adverse health outcomes. After 2011, some epidemiological studies have also been carried out to explore the direct relationship between consumption of UPF and MetS risk (3, 23–30), but the results remain controversial. So far, five published studies have reported that higher intake of UPF was associated with an increased risk of MetS (23, 25–29), while others showed a null association (3, 24, 30). For instance, during a median follow-up time of 6 years (IQR: 3.0–9.0), an analysis of China Health and Nutrition Survey (CHNS) showed that higher long-term UPF consumption was associated with an increased risk of MetS in Chinese adults (RR: 1.17, 95%CI: 1.01–1.35) (23). Similarly, in the Brazilian Longitudinal Study of Adult Health (ELSA-Brasil), Canhada et al., also found a positive association between consumption of UPF and the risk of MetS (RR: 1.19, 95%CI: 1.07–1.32) (25). In contrast, a recent prospective cohort study of 896 Brazil adults found no significant association between UPF consumption and risk for MetS (RR: 1.00, 95%CI: 0.99–1.01) (3). Indeed, previous meta-analyses have consistently shown a strong association between specific types of ultra-processed foods, such as soft drink, processed meat and risk of MetS (31, 32). Meanwhile, Lane et al. also published a systematic review and meta-analysis of observational studies reporting the association between consumption of ultraprocessed food and chronic non-communicable diseases (33). However, the above-mentioned meta-analysis regarding the relationship between UPF consumption and MetS risk was limited by a relatively limited number of studies available for inclusion at the time of publication (*n* = 4). Since then, several new epidemiological studies have also been published evaluating the association of UPF consumption with the risk of MetS (3, 23–25, 30). Furthermore, Lane et al.'s work has some limitations. For example, they only included four studies, which did not perform subgroup analyses to explore the potential sources of heterogeneity. Also, because the previous meta-analysis was based on four included studies, their results cannot be generalized to other populations. Therefore, we undertook a comprehensive systematic review and meta-analysis with the purpose to clarify the relationship between UPF consumption as defined by the NOVA framework and risk of MetS.

## Methods

### Search strategy

This systematic review and meta-analysis was performed in accordance with the Preferred Reporting Items for Systematic Reviews and Meta-Analyses guidelines (34). The protocol of the present study was not registered in PROSPERO. We carried out a comprehensive literature search, without any restrictions in time or language, up to March 2023 through the PubMed, ISI Web of Science, EBSCO and CNKI databases to identify all the published articles on the relationship between UPF consumption and risk of MetS. The following keywords or phrases, including those from the medical subject headings (MeSH) and non-MeSH terms, were utilized in this search: (“ultra-processed food” OR “ultraprocessed food” OR “UPF” OR “NOVA food classification”) AND (“metabolic syndrome” OR “MetS” OR “syndrome X”). Besides, the reference lists from the retrieved articles and published reviews were further searched for potentially relevant studies. The literature search was conducted by two authors (L.S. and C.-J.S). Disagreements were resolved by consensus after discussion with another author (Q.Z.). Our selection criteria was based on the PECOS (e.g. participant, exposure, comparison, outcome, and study design) framework, which is presented in Supplementary Table S1.

### Study selection

Two authors (C.-J. S and L.S.) independently screened and crosschecked each article from the literature search, and a third author (Q.Z.) was consulted to settle any discrepancies. After comprehensive screening all the titles and abstracts, the full-text versions of the articles were reviewed according to the inclusion and exclusion criteria of this meta-analysis. Studies were included in our analyses when they met all the following eligibility criteria: (1) were observational studies (cross-sectional, case-control or cohort studies); (2) were carried out in humans of any age; (3) UPF was considered as the main exposure of interest (according to the NOVA food classification system); (4) evaluated the association with MetS risk; (5) reported adjusted estimates of the RRs [e.g. hazard ratios (HRs) or odds ratios (ORs)] and 95%CIs for the link between UPF consumption and MetS risk. Where the original studies didn't provide sufficient data, the corresponding author of the study was contacted by email for additional information. Studies were excluded if they met one of the following criteria: (1) non-observational studies, e.g. reviews, editorials, case reports and conference letters; (2) animal, cell culture, and *in vitro* studies; (3) did not use the NOVA food classification system (assessed the only specific food or food groups, such as processed meat); (4) studies not reported as HRs, RRs or ORs with 95%CIs; (5) unrelated articles.

### Data extraction

Data were extracted by two independent authors (X.-Y. Z and J.-Y. Z) from the identified eligible studies, including first author's last name, year of publication, study design, study area, sample size, number of MetS cases, mean age or age range of participants, duration of follow-up for cohort studies, method of UPF assessment, adjustments for confounding factors, and effect sizes (ORs, HRs or RRs) for the relationship between UPF consumption and risk of MetS.

### Definition of ultra-processed food

In the NOVA food classification system, UPF is characterized by high intake of foods made up entirely or mostly from unhealthy components, including food products having high energy density, added sugar, salt, saturated and trans fats, and low amounts of dietary fiber, vitamins and minerals, e.g. pizza, instant noodles, hamburger and smoking meats (12).

### Quality assessment

The authors (L.S. and X.-Y. Z) independently assessed each included study's quality using the Newcastle-Ottawa Scale (NOS), which was designed for case-control and cohort studies (35). In the NOS checklist, scores ranged from 0 to 9 based on the eight items related to study selection (4 stars), comparability of participants (2 stars), and assessment of outcome/exposure of interest (3 stars). Finally, those studies with NOS scores ≥7 points were deemed as high quality, consistent with a previous meta-analysis (36). The assessment of the credibility of evidence was also carried out by using the NutriGrade scoring system (37). This tool comprises the eight items: (1) risk of bias, study quality, and study limitations (0 to 2 points); (2) precision (0 to 1 point); (3) heterogeneity (0 to 1 point); (4) directness (0 to 1 point); (5) publication bias (0 to 1 point); (6) funding bias (0 to 1 point); (7) effect size (0 to 2 points); and (8) dose-response (0 to 1 point). According to this NutriGrade score, ≥ 8 points, 6–7.99 points, 4–5.99 points and 0–3.99 points were defined as high, moderate, low and very low, respectively. Any discrepancies between two authors were resolved by a third author (Q.Z.) to reach a consensus.

### Data synthesis and statistical analyses

For the present analysis, we considered the HRs and ORs to be equivalent to RRs (36). In this study, data were measured as log RR with standard errors (SEs) by using the ORs, HRs, RRs and their corresponding 95%CIs. Random-effects or fixed-effects models were used to calculate the pooled RRs and 95% CIs. Heterogeneity among the included studies was tested by the Cochran's Q test and I-squared (I^2^) statistics. *P*-values of Cochran's Q test <0.10 and I^2^>50% were considered to show substantial heterogeneity among the included studies, and subsequently the random-effects models (DerSimonnian and Laird method) were used to summary the pooled RRs. Otherwise, the fixed-effects models were adopted (38). According to the I^2^ value, heterogeneity was classified as low (I^2^ ≤ 25%), moderate (25%~75%) and high (I^2^ ≥ 75%), respectively. In the case of significant between-study heterogeneity (I^2^ > 50%), the potential sources of heterogeneity across studies were explored using subgroup and sensitivity analyses. In our analyses, subgroup analyses were performed based on the study design (cohort or cross-sectional studies), exposure assessment (FFQ or 24h dietary recall), study quality (≥7 or <7), mean age (≥55 or <55), study area (developed countries or developing countries) and sample size (<5,000 or ≥5,000). Sensitivity analysis was performed, excluding one study removed at one time to confirm whether the results were robust or sensitive to the influence of individual study. Publication bias was assessed through examining the funnel plots, and statistical assessment of funnel plot asymmetry was quantified by Begg's or Egger's tests (39). When publication bias was detected, the trim and fill method was used to correct the results (40). All statistical analyses were conducted with STATA version 12.1 (College Station, Texas, USA). A 2-sided *P-*value ≤ 0.05 was considered as statistically significant unless otherwise specified.

## Results

### Overview of included studies for the systematic review

**Figure 1:**
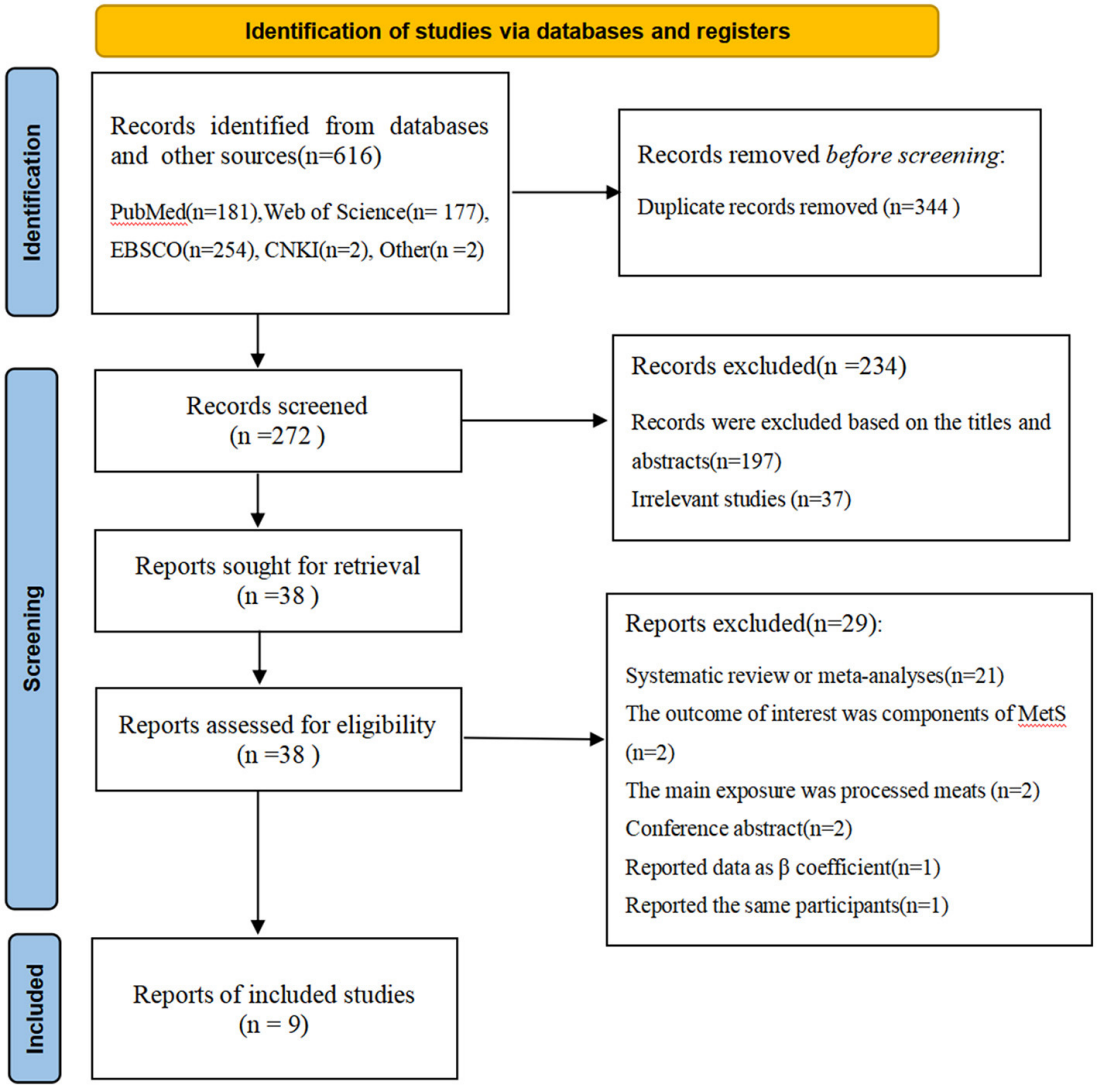
Flow chart of the process of the study selection.

Figure 1 shows flow chart of the process of the study selection. A total of 616 potentially relevant articles (181 for PubMed, 177 for Web of Science, 254 for EBSCO, 2 for CNKI, 2 for other sources) were retrieved during the initial literature search. After eliminating 344 duplicated articles, 272 articles remained. Subsequently, 197 articles were excluded based on the titles and abstracts and 37 irrelevant studies were also excluded. Of the remaining 38 full-text articles, 29 articles were excluded due to the following reasons: systematic review or meta-analyses (*n* = 21), the outcome of interest was components of metabolic syndrome (*n* = 2), the main exposure was processed meats (*n* = 2), conference abstract (*n* = 2), reported data as β coefficient (*n* = 1) and reported the same participants (*n* = 1). Finally, nine articles met the inclusion criteria and were included in our main analyses.

### Characteristics of the studies

The main characteristics of all included studies are outlined in Table 1. Altogether, nine articles with 23,500 participants and 6,192 MetS cases were included in this systematic review and meta-analysis. All these included studies were published in English between 2012 and 2023. Sample sizes ranged from 210 to 8065 participants. The age of participants ranged from ages 18 to above. The majority of the included studies were cross-sectional in design (24, 26–30), and only three were prospective cohort design (3, 23, 25). Among eligible studies, four of the included studies were conducted in Brazil (3, 24–26), one in Israel (29), one in United States (27), one in Lebanon (30), one in China (23), and one in Canada (28). The follow-up duration for cohort studies ranged from 8 to 29 years. Sample size ranged from 210 to 8,065. All included studies classified UPF intake according to the NOVA food classification systems (3, 23–30). Dietary data were collected using 24-h dietary recalls (23, 24, 27, 28) and FFQs (3, 25, 26, 29, 30), respectively. Overall, based on the NOS scores, eight of all the included studies were deemed as of high quality (3, 23–25, 27, 29, 30), and the remaining one was of low quality (26). The other characteristics of the included studies are described in Supplementary Table S2.

**Table 1:** Characteristics of the included studies on the association between consumption of ultra-processed food and risk of metabolic syndrome.

**References**	**Study design**	**Country**	**Sample size**	**Follow-up (years)**	**Mean age/age range**	**Exposure assessment**	**Outcome**	**Effect sizes OR/RR (95%CI)**	**Study quality**	**Adjustment**
Magalhães et al. (3)	Cohort	Brazil	896	12–16 y	37–39 y	FFQ	MetS	1.00 (0.99–1.01)	8	Sex, skin color, age, education, marital status, family income, alcohol consumption, smoking, level of physical activity and total caloric intake
Pan et al. (23)	Cohort	China	5,147	29	≥18 y	24-h dietary recall	MetS	1.17 (1.01–1.35)	9	Gender, age, education level, place of residence, regions, income level, smoking history, drinking status, metabolic equivalents, urbanicity, BMI, total energy intake, protein intake, fat intake, carbohydrate intake, and sodium intake
Barbosa et al. (24)	Cross-sectional	Brazil	895	-	19–55 y	24-h dietary recall	MetS	1.09 (0.89–1.32)	7	Age, race/skincolor, marital status, schooling, family participation in a government program, family income, employment status, food insecurity, smoking and health problems in the last 15 days, excess weight and neck circumference
Canhada et al. (25)	Cohort	Brazil	8,065	8 y	35–74 y	FFQ	MetS	1.19 (1.07–1.32)	9	Age, sex, center, race or color, income, school achievement, smoking, physical activity, alcohol, energy intake, and BMI
Tavares et al. (26)	Cross-sectional	Brazil	210	-	12–19 y	FFQ	MetS	2.49 (1.24–3.57)	6	Smoking, family hypertriacylglycerolaemia and energy intake
Martínez Steele et al. (27)	Cross-sectional	USA	6,385	-	≥20 y	24-h dietary recall	MetS	1.20 (1.07–1.35)	7	Sex, age group, race/ethnicity, ratio of family income to poverty and educational attainment, smoking status, physical activity, BMI (continuous).
Lavigne-Robichaud et al. (28)	Cross-sectional	Canada	811	-	≥18 y	24-h dietary recall	MetS	1.90 (1.14–3.17)	7	Age (continuous), sex, area of residence (coastal/inland), current smoker (yes/no), alcohol drinker (yes/no, except for the aHEI- 2010 model) and total dietary energy intake (kcal/d, continuous)
Ivancovsky-Wajcman et al. (29)	Cross-sectional	Israel	789	-	40–70 y	FFQ	MetS	1.88 (1.31–2.71)	7	Age, gender, BMI, saturate fatty acids and protein intake (% of total kcal), physical activity (hours/week), coffee (cups/day) and fibers (g/day)
Nasreddine et al. (30)	Cross-sectional	Lebanon	302	-	≥18 y	FFQ	MetS	1.11 (0.26–4.65)	6	Age, gender, marital status, area of residence, level of education, income, smoking status, physical activity, total energy intake and BMI.

USA, United States; CVD, cardiovascular disease; BMI, Body mass index; DGAI, Dietary Guidelines Adherence Score; WC, Waist circumference.

### Ultra-processed food intake and MetS risk

**Figure 2:**
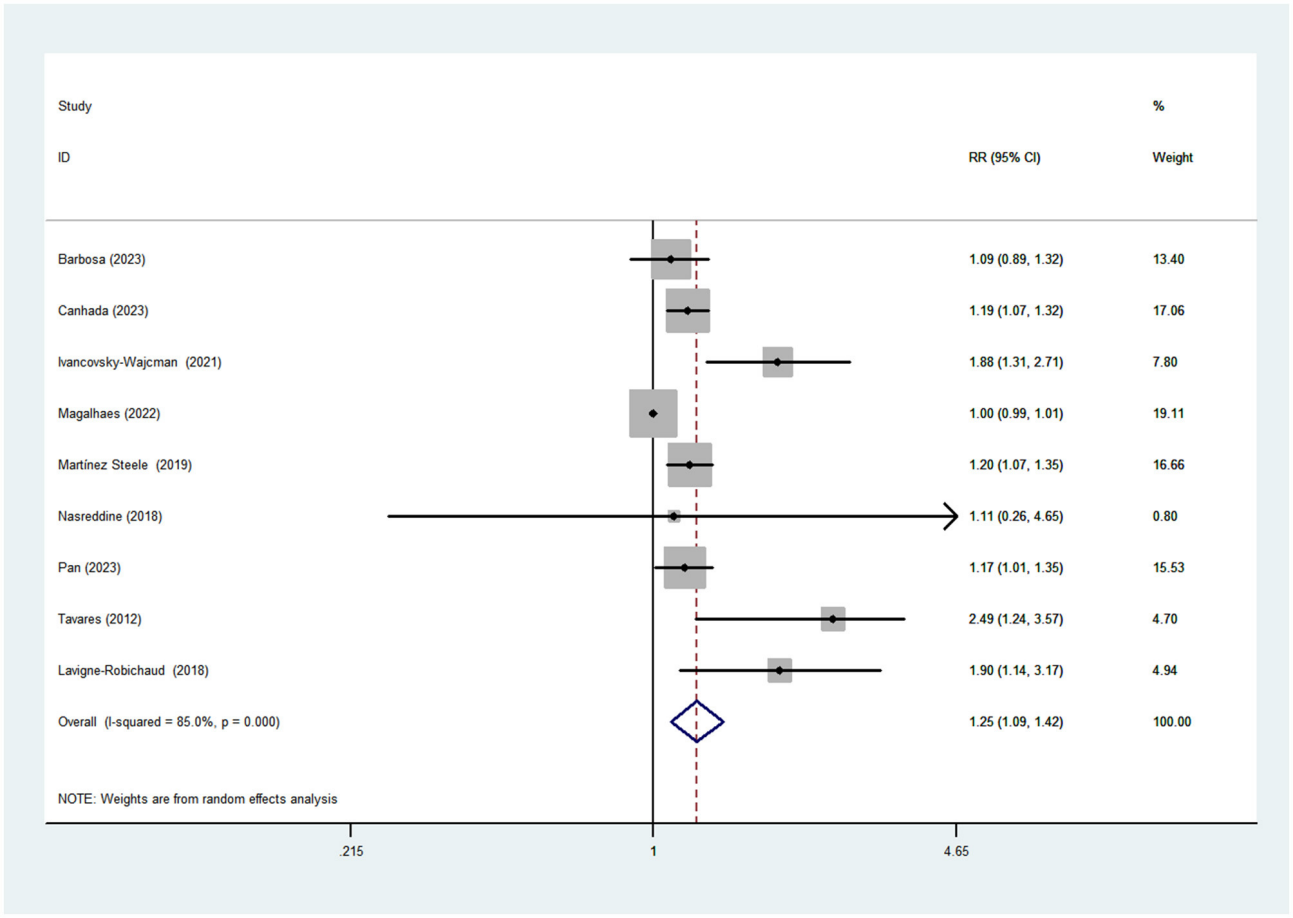
Forest plot of the association between consumption of UPF and MetS.

Nine studies (five cross-sectional and three cohort studies) including 6192 cases and 23500 participants were included in this meta-analysis that investigated the association between UPF consumption and MetS. Figure 2 shows obvious evidence of an increased risk of MetS in the highest compared with the lowest categories of UPF consumption (RR = 1.25; 95%CI: 1.09–1.42, *P* < 0.0001). The high heterogeneity was observed among the included studies (I^2^ = 85.0%; *P* < 0.0001), thus random-effects model was used to calculate the pooled RRs.

### Subgroup analyses

Given the significant heterogeneity of this meta-analysis (I^2^ = 85.0%, *P* < 0.0001), we performed subgroup analyses to find the potential sources of heterogeneity. The results of subgroup analyses were presented in Table 2. In this study, subgroup analyses were stratified basing on the study design (cohort or cross-sectional studies), exposure assessment (FFQ or 24 h dietary recall), study quality (≥7 or <7), mean age (≥55 or <55), study area (developed countries or developing countries) and sample size (<5,000 or ≥5,000). When we conducted analyses separately by study design, results showed a positive association between UPF intake and MetS risk in cross-sectional studies (RR = 1.47; 95%CI: 1.16–1.87, *P* = 0.002). However, there was evidence of heterogeneity between studies (*P* = 0.006; I^2^ = 69.3%). Meanwhile, there was no significant association between UPF intake and MetS risk in cohort studies (RR = 1.10; 95%CI: 0.96–1.27, *P* = 0.104), with more evidence of heterogeneity (*P* = 0.001; I^2^ = 86.5%). For exposure assessment, there was a significant association between UPF intake and risk of MetS (RR = 1.19; 95%CI: 1.07–1.31, *P* = 0.001) in 24-h dietary recall, with less evidence of heterogeneity (*P* = 0.257; I^2^ = 25.7%). In addition, we also found significant association between UPF intake and MetS risk in FFQ (RR = 1.33; 95%CI: 1.06–1.66, *P* = 0.014), and there was more heterogeneity (*P* < 0.0001; I^2^ = 88.0%). For mean age, the positive association between UPF intake and risk of MetS was observed in the subgroups of age <50 (RR = 1.31; 95%CI: 1.00–1.72, *P* = 0.053), and there was evidence of significant heterogeneity (*P* = 0.001; I^2^ = 78.0%). Moreover, we also observed the significant positive association between UPF intake and MetS risk in the subgroups of age ≥50 (RR = 1.23; 95%CI: 1.11–1.36, *P* < 0.0001), and there was evidence of heterogeneity (*P* = 0.113; I^2^ = 49.7%). For study area, there was a significant association between UPF intake and risk of MetS in developed countries (RR = 1.54; 95%CI: 1.08–2.22, *P* = 0.019) and the between-studies heterogeneity decreased from 85.0% to 74.3%. However, the statistical association was also observed between UPF intake and risk of MetS in developing countries (RR = 1.15; 95%CI: 1.01–1.33, *P* = 0.041). For sample size, we found a positive association between UPF intake and risk of MetS in sample size <5,000 (RR = 1.43; 95%CI: 1.08–1.90, *P* = 0.013). However, the heterogeneity was apparent (*P* < 0.0001, I^2^ = 83.2%). In contrast, we also found a significant association between UPF intake and MetS risk in sample size ≥5,000 (RR = 1.19; 95%CI: 1.11–1.27, *P* < 0.0001), and there was no heterogeneity (*P* = 0.965; I^2^ = 0.0%). For study quality, significant positive association was found between UPF intake and MetS risk in the subgroups of study quality ≥7 (RR = 1.20; 95%CI: 1.06–1.36, *P* = 0.005), and there was more heterogeneity (*P* < 0.0001, I^2^ = 85.7%). In addition, there was less evidence of heterogeneity in the subgroups of study quality <7 (*P* = 0.303; I^2^ = 5.6%), where significant positive association with risk of MetS was observed (RR = 2.22; 95%CI: 1.28–3.84, *P* = 0.004).

**Table 2:** Subgroup analyses for the association between UPF consumption and risk of MetS.

**Study characteristic**	**No. of studies**	**RR (95%CI)**	**Heterogeneity**	
			*I*^2^ **(%)**	***P***
All	9	1.25 (1.09–1.42)	85.0	<0.001
**Study design**				
Cross-sectional	6	1.47 (1.16–1.87)	69.3	0.006
Cohort	3	1.10 (0.96–1.27)	86.5	0.001
**Exposure assessment**				
FFQ	5	1.33 (1.06–1.66)	88.0	<0.001
24 h dietary recall	4	1.19 (1.07–1.31)	25.7	0.257
**Study quality**				
≥7	7	1.20 (1.06–1.36)	85.7	<0.001
<7	2	2.22 (1.28–3.84)	5.6	0.303
**Mean age**				
≥50	4	1.23 (1.11–1.36)	49.7	0.113
<50	5	1.31 (1.00–1.72)	78.0	0.001
**Study area**				
Developing countries	6	1.15 (1.01–1.33)	81.4	<0.001
Developed countries	3	1.54 (1.08–2.22)	74.3	0.021
**Sample size**				
≥5,000	3	1.19 (1.11–1.27)	0.0	0.965
<5,000	6	1.43 (1.08–1.90)	83.2	<0.001

UPF, Ultra-processed food; MetS, Metabolic syndrome; FFQ, Food frequency questionnaire; RR, relative risk; CI, confidence interval.

### Publication bias

As shown in Supplementary Figure 1, inspection of funnel plots revealed obvious evidence of asymmetry. Egger's test for publication bias had statistical significance (highest vs. lowest consumption: Egger's test: *P* = 0.001). However, in our analyses, Begg's test for publication bias was not statistically significant (highest vs. lowest level of UPF consumption: Begg's test: *P* = 0.917). When trim and fill was applied filling added no study to the funnel plot, indicating a low degree of asymmetry and no change in the overall effect.

### Sensitivity analysis

Based on the findings of sensitivity analysis (Supplementary Figure 2), a cohort study by Magalhãesas et al. was outside the limit, and might be the source of heterogeneity. When Magalhãesas et al.' study was excluded in the repeat analysis (Supplementary Figure 3), sensitivity analysis revealed a slight increase in the pooled RRs on the relationship between UPF consumption and risk of MetS (RR = 1.28; 95%CI: 1.14–1.45, *P* < 0.0001). In addition, the heterogeneity of included studies decreased from 85.0 to 59.2%.

### Quality assessment

The quality of included studies using NOS criteria is shown in Table 3. When included studies received a score of seven or higher, they would be deemed to be of relatively higher quality (3, 23–25, 27, 29, 30). Moreover, the remaining one articles was identified as low-quality (26). According to the NutriGrade score, the credibility of evidence was moderate considering studies that assessed the exposure with the NOVA food classification system (Table 4).

**Table 3:** Ultra-processed food consumption and risk of metabolic syndrome: assessment of study quality.

**Studies**	**Selection**				**Comparability**		**Outcome**			**Score**
	**1**	**2**	**3**	**4**	**5A**	**5B**	**6**	**7**	**8**	
**Cohort**										
Magalhães et al. (3)	^*^	^*^	^*^	^*^	^*^		^*^	^*^	^*^	8
Pan et al. (23)	^*^	^*^	^*^	^*^	^*^	^*^	^*^	^*^	^*^	9
Canhada et al. (25)	^*^	^*^	^*^	^*^	^*^	^*^	^*^	^*^	^*^	9
**Cross-sectional**										
Barbosa et al. (24)	^*^	^*^	^*^	^*^	^*^		^*^	^*^		7
Tavares et al. (26)	^*^	^*^	^*^		^*^		^*^	^*^		6
Martínez Steele et al. (27)	^*^	^*^	^*^	^*^	^*^		^*^	^*^		7
Lavigne-Robichaud et al. (28)	^*^	^*^	^*^	^*^	^*^		^*^	^*^		7
Ivancovsky-Wajcman et al. (29)	^*^	^*^	^*^	^*^	^*^		^*^	^*^		7
Nasreddine et al. (30)	^*^	^*^	^*^		^*^		^*^	^*^		6

* For case-control studies, 1 indicates cases independently validated; 2, cases are representative of population; 3, community controls; 4, controls have no history of blood pressure disease; 5A, study controls for age; 5B, study controls for additional factor(s); 6, ascertainment of exposure by blinded interview or record; 7, same method of ascertainment used for cases and controls; and 8, non response rate the same for cases and controls. For cohort studies, 1 indicates exposed cohort truly representative; 2, non exposed cohort drawn from the same community; 3, ascertainment of exposure; 4, outcome of interest not present at start; 5A, cohorts comparable on basis of age; 5B, cohorts comparable on other factor(s); 6, quality of outcome assessment; 7, follow-up long enough for outcomes to occur; and 8, complete accounting for cohorts.

**Table 4:** Credibility of evidence using NutriGrade tool for association between UPF consumption and MetS.

	**UPF consumption evaluated by NOVA classification system**
**NutriGrade items**	
Risk of bias^a^	2
Precision^b^	1
Indirectness	0
Heterogeneity^c^	0.5
Publication bias^d^	0.5
Effect size^e^	1
Dose-response	0
Funding bias	1
Total score	6
Credibility of evidence	Moderate

UPF, Ultra-processed food; MetS, Metabolic syndrome.

a Risk of bias was based on the Newcastle-Ottawa Scale, where ≥7 = 2 points; 4–6.9 = 1 point; and 0–3.9 = 0 points.

b Precision is 1 point if the number of events ≥500 and the 95% CI excludes the null value; precision is 0 points if the number of events <500 or number of events ≥500, but 95% CI includes the null value (e.g., CI includes RR of 1.0) and 95% CI fails to exclude an important benefit (RR of 0.8) or harm (RR of 1.2).

c When I^2^ was <40% or I^2^ was ≥40% but the source of heterogeneity was found by subgroup analysis 1 point was assigned; otherwise, 0 points were assigned.

d Based on the funnel plots, Egger or Begg's test. <5 studies = 0 points; no evidence for publication bias with test or plot (≥10 studies) = 1 point.

e If the RR or HR <0.80–0.50 and >1.20–2.00, respectively, 1 point is assigned and the corresponding test is statistically significant; if the RR or HR <0.50 and >2.00, respectively, 0 points are assigned and the corresponding test is statistically significant.

## Discussion

In the present study, the pooled results illustrated that high consumption of UPF was significantly associated with an increased risk of MetS. Nonetheless, the results of this meta-analysis must be interpreted with caution due to the high heterogeneity among the included studies. Moreover, subgroup analyses showed the positive association between high consumption of UPF and risk of MetS was more robust in cross-sectional studies and the subgroups of study quality <7. Likewise, the results of sensitivity analysis indicated that Magalhãesas et al.' study might be the source of heterogeneity. To the authors' knowledge, this is the first comprehensive systematic review and meta-analysis to assess the relationship between consumption of UPF and MetS risk. Our findings confirm the positive association results of previous studies and add to the growing evidence for the role of UPF consumption in diet-related chronic diseases, including MetS.

With economic development and changes in lifestyle, the prevalence of MetS is increasing around the world (2). It is reported that the standardized prevalence of MetS was 31.1% in 2015–2017, and approximately a third of adults have MetS in China (7). Given the high prevalence and burden on public health, more attention is now needed to prevent the occurrence of MetS. As we all know, dietary factors, as a component of lifestyle, play a key role in the prevention of MetS (8). Over the past decades, the food supply industries have increased the commercialization of UPF, and usual diets have also shifted toward the consumption of UPF, characterized by high in energy density, added sugar, saturated and trans-fats, as well as lower in fiber (41). In recent decade, the global consumption of UPF has increased rapidly, contributing about 25%~60% of total daily energy intake in some high- and middle-income countries (19). However, there are considerable differences in UPF consumption between developing and developed countries (42, 43). For example, UPF consumption has already accounted for more than 50% of total energy intake in countries such as United States, Canada and United kingdom (44–46). By contrast, the median contribution of UPF intake to the total daily energy was 10.5% in China (47). Although the overall consumption of UPF in China is currently lower than that observed in some high-income countries, the increased trend was dramatic, especially in highly urbanized areas (48). According to China's most recent census in 2020, the proportion of individuals aged 65 years and above in China has reached nearly 14%, indicating that China is becoming an elderly society (49). UPF consumption is associated with poorer diet quality (i.e. low dietary fiber, fruits and vegetables intake), which can lead to frailty. A recent cross-sectional study conducted by Zupo et al., offered the evidence of food processing contribution to poor nutrition in the aging population (50). Hence, the influence of UPF consumption on chronic diseases has garnered considerable attention from researchers. Until now, some previous observational studies have shown that higher consumption of UPF is significantly associated with adverse health outcomes, such as the increased risks for obesity, hypertension, diabetes, metabolic syndrome and cancers (16, 20–22). However, epidemiological evidence regarding the effect of consumption of UPF on the risk of MetS is limited and inconclusive (13, 23–30). In this study, we found a significant positive relationship between UPF intake and MetS risk, although there was evidence of high heterogeneity across studies (I^2^ = 85.0%; *P* < 0.001). This is in agreement with findings from some previous studies reporting that high UPF consumption is associated with an increased risk of MetS (23, 25, 27–29). In the China Health and Nutrition Survey (CHNS), Pan et al. reported that higher long-term UPF consumption was associated with an increased risk of MetS in Chinese adults (RR = 1.17; 95% CI: 1.01–1.35) over a follow-up period of 29 years (23). Similarly, a recent study from the Brazilian longitudinal study of adult health also showed that higher consumption of UPFs was associated with an increased risk of MetS (RR = 1.19; 95%CI: 1.07- 1.32) (25). However, contrary to our finding, a recent cross-sectional study conducted by Barbosa et al., showed no significant relationship between UPF consumption and MetS risk (RR = 1.09, 95%CI: 0.89–1.32) (24). The differences in assessment of UPF, amounts and types of UPF of different populations, and definitions of UPF consumption levels, at least in part, explain the discrepant results between the different studies (23). Moreover, the inconsistency may also result from the significant difference in the statistical power. Previous studies undertaken in Lebanon and Brazil have documented a small number of MetS, which results in the limited power (26, 30). Furthermore, it is worth noticing that these inconsistent findings may be attributed to the differences in sociodemographic factors, such as age, race, and income, which have been found to be associated with UPF consumption (12). Researchers have proposed several potential plausible mechanisms that may explain the observed positive association between UPF consumption and risk of MetS, although conflicting results were reported. First, UPFs tend to be energy dense and often have high amounts of saturated fat, trans-fat and added sugars. Previous studies have demonstrated that excessive consumption of saturated fat and added sugars are associated with higher risk of MetS (51). Second, the detrimental effect of UPF consumption on MetS may also partly be attributed to lower intakes of minimally processed foods such as whole grains, vegetables, fruits, which are shown to be inversely associated with risk of MetS (52). Third, beyond the poor nutritional composition, other components common present in UPF, such as emulsifiers and artificial sweeteners have been implicated in changes in gut microbiota, glucose intolerance and insulin resistance, which could lead to the progression of MetS (53–55). Fourth, UPF may be contaminated with packaging contact materials, such as phthalates and bisphenol A, are involved in endocrine disruption and insulin resistance (56). A previous meta-analysis of 33 epidemiological studies on Bisphenol A and risk of cardiometabolic disorders showed that higher concentration of Bisphenol A was associated with an increased risk of cardiometablic outcomes (57). Finally, food processing (particularly heat treatments) is largely associated with the loss of physical and structural characteristics of the food substrate, and has been reported to be associated with lower satiety potential and higher glycemic responses (58). Given the above, these plausible mechanisms could explain the positive association between UPF consumption and risk of MetS.

In our analyses, it is important to point out that there was evidence for high between-study heterogeneity among all included studies (I^2^ = 85.0%, *P* < 0.0001). Although between-study heterogeneity is common in meta-analysis (59), exploring the potential sources of heterogeneity is the essential. Thus, we performed subgroup analyses based on study design (cohort or cross-sectional studies), exposure assessment (FFQ or 24 h dietary recall), study quality (≥7 or <7), mean age (≥55 or <55), study area (developed countries or developing countries) and sample size (<5,000 or ≥5,000) to address sources of heterogeneity. The results of subgroup analyses showed that heterogeneity might be mainly due to the difference in study design and exposure assessment. When the results were stratified by exposure assessment, the heterogeneity among included studies decreased from 85.0 to 25.7%. There are several possible explanations for the high heterogeneity. First, different levels of UPF consumption in included studies may explain, to some extent, the high between-study heterogeneity. Second, six of the included studies were cross-sectional in design. Given the observational nature of included studies, we cannot assume the causality of the observed association. Likewise, the results may be susceptible to recall bias, resulting from dietary survey methods (i.e. FFQs and 24-h dietary recalls) in the observational studies. Third, considering that UPF consumption varied across different populations, and that definitions of UPF consumption levels varied in different studies, despite the RRs or ORs were all from the highest category (taking the lowest category as the reference), different populations and studies may have different definitions of UPF consumption levels, thereby causing the substantial heterogeneity. Fourth, different models used to control potential confounding variables in included studies may explain the heterogeneity observed in our analyses. There was an inconsistent adjustment for potential confounding variables in the included studies. As a consequence, it is inevitable that we have high levels of heterogeneity when combining studies. Finally, the considerable heterogeneity persisted in subgroup analyses, indicating the presence of other unknown confounding factors.

## Strengths and limitations

This study has several strengths and limitations. First, to our knowledge, this is the first comprehensive systematic review and meta-analysis to evaluate the relationship between consumption of UPF and the risk of MetS. Our findings add to the growing evidence for the impact of UPF consumption on MetS and help inform public policy for the prevention and management of MetS. Second, a rigorous selection of articles was conducted based on the predetermined inclusion criteria, with the inclusion only of studies in which the classification of UPFs faithfully followed the characteristics proposed by the NOVA system. Third, MetS cases were ascertained through medical records, reducing the risk of misclassification. Fourth, the quality of the included studies was moderate to high, and the reported RRs were multivariate and adjusted for some known confounders, such as sex, age, physical activity and total energy intake. Meanwhile, we also performed subgroup and sensitivity analyses to explore the potential sources of heterogeneity. Fifth, no signs of publication bias were evident in the funnel plot, and the Begg's test for publication bias was non-significant. Despite the above-mentioned strengths, this study also has some limitations that should be acknowledged. First, although our findings show a positive relationship between UPF consumption and MetS risk, the majority of included studies are cross-sectional in design, which are limited by the potential for reverse causality. Second, five of the included studies used the FFQs that were not specifically designed to assess the NOVA classification groups, which might have led to an under- or over-estimation of the size of the observed associations. Likewise, information bias, as a consequence of self-reported data on dietary intake, might have occurred. Third, even though all of included studies in this meta-analysis have adjusted for a wide range of important confounders, residual confounding from unmeasured factors cannot be completely ruled out. Also, there was also an inconsistent adjustment for potential confounders in the all included studies. Consequently, the data included in this meta-analysis might suffer from differing degrees of completeness and accuracy. Fourth, high heterogeneity was found in this meta-analysis, which might have distorted the reliability of our results. Although we performed subgroup and sensitivity analyses to explore the potential sources of heterogeneity, we were unable to adequately ascertain and explain the sources of inter-study heterogeneity. Finally, given the limited number of included studies and the fact that most of them were performed in developing countries, caution is advised in the interpretation and extrapolation of our findings.

## Conclusion

In summary, findings from this study suggest that higher consumption of UPF is significantly related to an increased risk of developing MetS. Our findings add valuable evidence to the existing literature showing a positive relationship between UPF consumption and risk of MetS, and highlight the importance of limiting UPF consumption in decreasing the modifiable burden of MetS. Thus, active discouragement of UPF consumption should be considered as part of MetS prevention strategies. Considering the high level of evidence provided in the included studies, more well-designed prospective studies, particularly in different geographic regions and settings, are warranted to further confirm these findings.

## Data availability statement

The original contributions presented in the study are included in the article/Supplementary material, further inquiries can be directed to the corresponding author.

## Author contributions

CS conceived and designed the systematic review, meta-analysis, and interpreted the results. XZ and JZ acquired the data. LS and QZ performed the statistical analysis and obtained the funding. LS conceived the idea and drafted this manuscript. All authors critically revised the manuscript for important intellectual content. All authors contributed to the article and approved the submitted version.

## References

[1] Alberti KG Eckel RH Grundy SM Zimmet PZ Cleeman JI Donato KA . Harmonizing the metabolic syndrome: a joint interim statement of the International Diabetes Federation Task Force on Epidemiology and Prevention; National Heart, Lung, and Blood Institute; American Heart Association; World Heart Federation; International Atherosclerosis Society; and International Association for the Study of Obesity. Circulation. (2009) 120:1640–5. 10.1161/CIRCULATIONAHA.109.192644 19805654

[2] Aguilar M Bhuket T Torres S Liu B Wong RJ. Prevalence of the metabolic syndrome in the United States, 2003-2012. JAMA. (2015) 313:1973–4. 10.1001/jama.2015.4260 25988468

[3] Magalhães EIDS de Oliveira BR Rudakoff LCS de Carvalho VA Viola PCAF Arruda SPM . Sex-dependent effects of the intake of NOVA classified ultra-processed foods on syndrome metabolic components in Brazilian adults. Nutrients. (2022) 14:3126. 10.3390/nu14153126 35956300 PMC9370159

[4] He Y Li Y Bai G Zhang J Fang Y Zhao L . Prevalence of metabolic syndrome and individual metabolic abnormalities in China, 2002-2012. Asia Pac J Clin Nutr. (2019) 28:621–33. 10.6133/apjcn.201909_28(3).0023 31464410

[5] Moore JX Chaudhary N Akinyemiju T. Metabolic syndrome prevalence by race/ethnicity and sex in the united states, national health and nutrition examination survey, 1988-2012. Prev Chronic Dis. (2017) 14:E24. 10.5888/pcd14.160287 28301314 PMC5364735

[6] Saklayen MG. The global epidemic of the metabolic syndrome. Curr Hypertens Rep. (2018) 20:12. 10.1007/s11906-018-0812-z 29480368 PMC5866840

[7] Yao F Bo Y Zhao L Li Y Ju L Fang H . Prevalence and influencing factors of metabolic syndrome among adults in China from 2015 to 2017. Nutrients. (2021) 13:4475. 10.3390/nu13124475 34960027 PMC8705649

[8] Karimi G Heidari Z Firouzi S Haghighatdoost F. A systematic review and meta-analysis of the association between fish consumption and risk of metabolic syndrome. Nutr Metab Cardiovasc Dis. (2020) 30:717–29. 10.1016/j.numecd.2020.02.001 32127332

[9] Luan D Wang D Campos H Baylin A. Red meat consumption and metabolic syndrome in the costa rica heart study. Eur J Nutr. (2020) 59:185–93. 10.1007/s00394-019-01898-6 30649594

[10] Lee M Lim M Kim J. Fruit and vegetable consumption and the metabolic syndrome: a systematic review and dose-response meta-analysis. Br J Nutr. (2019) 122:723–33. 10.1017/S000711451900165X 31514758

[11] Jang H Park K. Omega-3 and omega-6 polyunsaturated fatty acids and metabolic syndrome: A systematic review and meta-analysis. Clin Nutr. (2020) 39:765–73. 10.1016/j.clnu.2019.03.032 31010701

[12] Monteiro CA Cannon G Levy RB Moubarac JC Louzada ML Rauber F . Ultra-processed foods: what they are and how to identify them. Public Health Nutr. (2019) 22:936–41. 10.1017/S1368980018003762 30744710 PMC10260459

[13] Souza TN Andrade GC Rauber F Levy RB da Costa Louzada ML. Consumption of ultra-processed foods and the eating location: can they be associated? Br J Nutr. (2022) 128:1587–94. 10.1017/S0007114521004992 34915943

[14] Fiolet T Srour B Sellem L Kesse-Guyot E Allès B Méjean C . Consumption of ultra-processed foods and cancer risk: results from NutriNet-Santé prospective cohort. BMJ. (2018) 360:k322. 10.1136/bmj.k322 29444771 PMC5811844

[15] De Nucci S Zupo R Castellana F Sila A Triggiani V Lisco G . Public Health Response to the SARS-CoV-2 pandemic: concern about ultra-processed food consumption. Foods. (2022) 11:950. 10.3390/foods11070950 35407037 PMC8997472

[16] Rauber F Chang K Vamos EP da Costa Louzada ML Monteiro CA Millett C Levy RB. Ultra-processed food consumption and risk of obesity: a prospective cohort study of UK Biobank. Eur J Nutr. (2021) 60:2169–80. 10.1007/s00394-020-02367-1 33070213 PMC8137628

[17] Levy RB Rauber F Chang K Louzada MLDC Monteiro CA Millett C . Ultra-processed food consumption and type 2 diabetes incidence: a prospective cohort study. Clin Nutr. (2021) 40:3608–14. 10.1016/j.clnu.2020.12.018 33388205

[18] Li M Shi Z. Ultra-processed food consumption associated with incident hypertension among Chinese adults-results from china health and nutrition survey 1997-2015. Nutrients. (2022) 14:4783. 10.3390/nu14224783 36432470 PMC9692874

[19] Srour B Fezeu LK Kesse-Guyot E Allès B Méjean C Andrianasolo RM . Ultra-processed food intake and risk of cardiovascular disease: prospective cohort study (NutriNet-Santé). BMJ. (2019) 365:l1451. 10.1136/bmj.l1451 31142457 PMC6538975

[20] Delpino FM Figueiredo LM Bielemann RM da Silva BGC Dos Santos FS Mintem GC . Ultra-processed food and risk of type 2 diabetes: a systematic review and meta-analysis of longitudinal studies. Int J Epidemiol. (2022) 51:1120–41. 10.1093/ije/dyab247 34904160

[21] Taneri PE Wehrli F Roa-Díaz ZM Itodo OA Salvador D Raeisi-Dehkordi H . Association between ultra-processed food intake and all-cause mortality: a systematic review and meta-analysis. Am J Epidemiol. (2022) 191:1323–35. 10.1093/aje/kwac039 35231930

[22] Wang M Du X Huang W Xu Y. Ultra-processed foods consumption increases the risk of hypertension in adults: a systematic review and meta-analysis. Am J Hypertens. (2022) 35:892–901. 10.1093/ajh/hpac069 35750049

[23] Pan F Wang Z Wang H Zhang J Su C Jia X . Association between ultra-processed food consumption and metabolic syndrome among adults in China-results from the China health and nutrition survey. Nutrients. (2023) 15:752. 10.3390/nu15030752 36771458 PMC9921592

[24] Barbosa LB Vasconcelos NBR Dos Santos EA Dos Santos TR Ataide-Silva T Ferreira HDS. Ultra-processed food consumption and metabolic syndrome: a cross-sectional study in Quilombola communities of Alagoas, Brazil. Int J Equity Health. (2023) 22:14. 10.1186/s12939-022-01816-z 36650595 PMC9847020

[25] Canhada SL Vigo Á Luft VC Levy RB Alvim Matos SM Del Carmen Molina M . Ultra-processed food consumption and increased risk of metabolic syndrome in adults: the ELSA-Brasil. Diabetes Care. (2023) 46:369–76. 10.2337/dc22-1505 36516280 PMC9887627

[26] Tavares LF Fonseca SC Garcia Rosa ML Yokoo EM. Relationship between ultra-processed foods and metabolic syndrome in adolescents from a Brazilian Family Doctor Program. Public Health Nutr. (2012) 15:82–7. 10.1017/S1368980011001571 21752314

[27] Martínez Steele E Juul F Neri D Rauber F Monteiro CA. Dietary share of ultra-processed foods and metabolic syndrome in the US adult population. Prev Med. (2019) 125:40–8. 10.1016/j.ypmed.2019.05.004 31077725

[28] Lavigne-Robichaud M Moubarac JC Lantagne-Lopez S Johnson-Down L Batal M Laouan Sidi EA . Diet quality indices in relation to metabolic syndrome in an Indigenous Cree (Eeyouch) population in northern Québec, Canada. Public Health Nutr. (2018) 21:172–80. 10.1017/S136898001700115X 28683844 PMC10260753

[29] Ivancovsky-Wajcman D Fliss-Isakov N Webb M Bentov I Shibolet O Kariv R . Ultra-processed food is associated with features of metabolic syndrome and non-alcoholic fatty liver disease. Liver Int. (2021) 41:2635–45. 10.1111/liv.14996 34174011

[30] Nasreddine L Tamim H Itani L Nasrallah MP Isma'eel H Nakhoul NF . A minimally processed dietary pattern is associated with lower odds of metabolic syndrome among Lebanese adults. Public Health Nutr. (2018) 21:160–71. 10.1017/S1368980017002130 28965534 PMC5729841

[31] Kim Y Je Y. Meat consumption and risk of metabolic syndrome: results from the Korean population and a meta-analysis of observational studies. Nutrients. (2018) 10:390. 10.3390/nu10040390 29565803 PMC5946175

[32] Muñoz-Cabrejas A Guallar-Castillón P Laclaustra M Sandoval-Insausti H Moreno-Franco B. Association between sugar-sweetened beverage consumption and the risk of the metabolic syndrome: a systematic review and meta-analysis. Nutrients. (2023) 15:430. 10.3390/nu15020430 36678301 PMC9912256

[33] Lane MM Davis JA Beattie S Gómez-Donoso C Loughman A O'Neil A . Ultraprocessed food and chronic noncommunicable diseases: a systematic review and meta-analysis of 43 observational studies. Obes Rev. (2021) 22:e13146. 10.1111/obr.13146 33167080

[34] Page MJ McKenzie JE Bossuyt PM Boutron I Hoffmann TC Mulrow CD . The PRISMA 2020 statement: an updated guideline for reporting systematic reviews. BMJ. (2021) 372:n71. 10.1136/bmj.n71 33782057 PMC8005924

[35] Stang A. Critical evaluation of the Newcastle-Ottawa scale for the assessment of the quality of nonrandomized studies in meta-analyses. Eur J Epidemiol. (2010) 25:603–5. 10.1007/s10654-010-9491-z 20652370

[36] Shu L Huang YQ Zhang XY Zheng PF Zhu Q Zhou JY. Adherence to the dietary approaches to stop hypertension diet reduces the risk of breast cancer: a systematic review and meta-analysis. Front Nutr. (2023) 9:1032654. 10.3389/fnut.2022.1032654 36698472 PMC9868726

[37] Schwingshackl L Knüppel S Schwedhelm C Hoffmann G Missbach B Stelmach-Mardas M . Perspective: nutrigrade: a scoring system to assess and judge the meta-evidence of randomized controlled trials and cohort studies in nutrition research. Adv Nutr. (2016) 7:994–1004. 10.3945/an.116.013052 28140319 PMC5105044

[38] Higgins JP Thompson SG Deeks JJ Altman DG. Measuring inconsistency in meta-analyses. BMJ. (2003) 327:557–5560. 10.1136/bmj.327.7414.557 12958120 PMC192859

[39] Begg CB Mazumdar M. Operating characteristics of a rank correlation test for publication bias. Biometrics. (1994) 50:1088–101. 10.2307/2533446 7786990

[40] Duval S Tweedie R. Trim and fill: a simple funnel-plot-based method of testing and adjusting for publication bias in meta-analysis. Biometrics. (2000) 56:455–63. 10.1111/j.0006-341X.2000.00455.x 10877304

[41] Kliemann N Rauber F Bertazzi Levy R Viallon V Vamos EP Cordova R . Food processing and cancer risk in Europe: results from the prospective EPIC cohort study. Lancet Planet Health. (2023) 7:e219–32. 10.1016/S2542-5196(23)00021-9 36889863 PMC10009757

[42] Baker P Friel S. Food systems transformations, ultra-processed food markets and the nutrition transition in Asia. Global Health. (2016) 12:80. 10.1186/s12992-016-0223-3 27912772 PMC5135831

[43] Monteiro CA Moubarac JC Cannon G Ng SW Popkin B. Ultra-processed products are becoming dominant in the global food system. Obes Rev. (2013) 14 (Suppl. 2):21–8. 10.1111/obr.12107 24102801

[44] Baraldi LG Martinez Steele E Canella DS Monteiro CA. Consumption of ultra-processed foods and associated sociodemographic factors in the USA between 2007 and 2012: evidence from a nationally representative cross-sectional study. BMJ Open. (2018) 8:e020574. 10.1136/bmjopen-2017-020574 29525772 PMC5855172

[45] Juul F Vaidean G Lin Y Deierlein AL Parekh N. Ultra-processed foods and incident cardiovascular disease in the Framingham offspring study. J Am Coll Cardiol. (2021) 77:1520–31. 10.1016/j.jacc.2021.01.047 33766258

[46] Moubarac JC Batal M Louzada ML Martinez Steele E Monteiro CA. Consumption of ultra-processed foods predicts diet quality in Canada. Appetite. (2017) 108:512–20. 10.1016/j.appet.2016.11.006 27825941

[47] Zhang S Gan S Zhang Q Liu L Meng G Yao Z . Ultra-processed food consumption and the risk of non-alcoholic fatty liver disease in the Tianjin Chronic Low-grade Systemic Inflammation and Health Cohort Study. Int J Epidemiol. (2022) 51:237–49. 10.1093/ije/dyab174 34528679

[48] Li M Shi Z. Association between ultra-processed food consumption and diabetes in Chinese adults-results from the China health and nutrition survey. Nutrients. (2022) 14:4241. 10.3390/nu14204241 36296925 PMC9609918

[49] Han X Wei C Cao GY. Aging, generational shifts, and energy consumption in urban China. Proc Natl Acad Sci U S A. (2022) 119:e2210853119. 10.1073/pnas.2210853119 36067298 PMC9478673

[50] Zupo R Donghia R Castellana F Bortone I De Nucci S Sila A . Ultra-processed food consumption and nutritional frailty in older age. Geroscience. (2023). 10.1007/s11357-023-00753-1 [Epub ahead of print]. 36826622 PMC10651811

[51] Martínez-González MÁ Martín-Calvo N. The major European dietary patterns and metabolic syndrome. Rev Endocr Metab Disord. (2013) 14:265–71. 10.1007/s11154-013-9264-6 23979531

[52] Tian Y Su L Wang J Duan X Jiang X. Fruit and vegetable consumption and risk of the metabolic syndrome: a meta-analysis. Public Health Nutr. (2018) 21:756–65. 10.1017/S136898001700310X 29151369 PMC10260986

[53] Suez J Korem T Zeevi D Zilberman-Schapira G Thaiss CA Maza O . Artificial sweeteners induce glucose intolerance by altering the gut microbiota. Nature. (2014) 514:181–6. 10.1038/nature13793 25231862

[54] Dabke K Hendrick G Devkota S. The gut microbiome and metabolic syndrome. J Clin Invest. (2019) 129:4050–7. 10.1172/JCI129194 31573550 PMC6763239

[55] Chassaing B Koren O Goodrich JK Poole AC Srinivasan S Ley RE . Dietary emulsifiers impact the mouse gut microbiota promoting colitis and metabolic syndrome. Nature. (2015) 519:92–6. 10.1038/nature14232 25731162 PMC4910713

[56] Stojanoska MM Milosevic N Milic N Abenavoli L. The influence of phthalates and bisphenol A on the obesity development and glucose metabolism disorders. Endocrine. (2017) 55:666–81. 10.1007/s12020-016-1158-4 27822670

[57] Rancière F Lyons JG Loh VH Botton J Galloway T Wang T . Bisphenol A and the risk of cardiometabolic disorders: a systematic review with meta-analysis of the epidemiological evidence. Environ Health. (2015) 14:46. 10.1186/s12940-015-0036-5 26026606 PMC4472611

[58] Fardet A Méjean C Labouré H Andreeva VA Feron G. The degree of processing of foods which are most widely consumed by the French elderly population is associated with satiety and glycemic potentials and nutrient profiles. Food Funct. (2017) 8:651–8. 10.1039/C6FO01495J 28106215

[59] Li Y. Association between fruit and vegetable intake and risk for glioma: a meta-analysis. Nutrition. (2014) 30:1272–8. 10.1016/j.nut.2014.03.027 25194962

