# Supplementary material for: Ultra-processed food consumption and increased risk of metabolic syndrome: a systematic review and meta-analysis of observational studies
Source: Front Nutr. 2023 Jun 9;10:1211797. doi: 10.3389/fnut.2023.1211797 (PMC10288143; doi:10.3389/fnut.2023.1211797)
Supplement: Supplementary file 1 [file Table_1.doc]

Table S1. PECOS criteria for inclusion and exclusion of studies

| **Population** | **General population** |
| --- | --- |
| Exposure | Ultra-processed food consumption |
| Comparison | Highest vs. lowest categories of exposure |
| Outcomes | Metabolic syndrome |
| Study design | Cross-sectional or case-control or cohort studies |

PECOS, participant, exposure, comparison, outcome, and study design
