# Supplementary material for: Ultra-processed food consumption and increased risk of metabolic syndrome: a systematic review and meta-analysis of observational studies
Source: Front Nutr. 2023 Jun 9;10:1211797. doi: 10.3389/fnut.2023.1211797 (PMC10288143; doi:10.3389/fnut.2023.1211797)
Supplement: Supplementary file 2 [file Table_2.doc]

**Table S2. Other characteristics of the included studies**

| Author, year | Ultra-processed food assessment method | Outcome | Effect sizes  OR/RR/PR(95%CI) |
| --- | --- | --- | --- |
| Magalhães et al. 2022 (3) | FFQ/NOVA food classification: in natura or minimally processed foods, processed culinary ingredients, and processed foods and ultraprocessed foods. | The criterion for diagnosing MetS was considered tobe a change in at least three components according to parameters established by the Joint Interim Statement (JIS) which are as follows: WC ≥ 90 cm for men and ≥80 cm for  women; triglycerides ≥ 150 mL/dL or use of antilipemic medicine; HDL-c < 40 mg/dL for men and <50 mg/dL for women or use of antilipemic medication; systolic ≥ 130 mmHg or diastolic arterial pressure ≥ 85 mmHg or use of antihypertensive medication; and fasting blood sugar ≥ 100 mg/dL or use of antihyperglycemic medication. | RR:1.00; 95%CI:0.99~1.01 |
| Pan et al.2023(23) | 24h dietary recall/NOVA food classification:unprocessed or minimally processed foods, processed culinary ingredients, processed foods and ultra-processed foods; UPF mainly includes the following food items, sugar-sweetened beverages (SSBs), packaged snacks, sweet, ice cream, chocolate, mass-produced packaged breads, cakes, desserts, biscuits, pastries, pre-prepared pies, pizza dishes,hot dogs,and sausages and other reconstituted meat products | MetS is defined using the National Cholesterol Education Program Adult Treatment Panel III (NCEP ATP III) criteria.If at least three out of five of the following components were present, the person was determined to have MetS:(1)central obesity:waist circumference(WC)≥90 cm (men) and ≥80cm (women); (2) raised triglycerides(TG):≥150mg/dL or relevant specific treatment for hyper-  lipidemia; (3) reduced high-density lipoprotein cholesterol (HDL-C): < 1.0 mmol/L (men) and 1.3 mmol/L (women); (4) raised blood pressure: systolic blood pressure (SBP) ≥ 130 mmHg or diastolic blood pressure (DBP)≥85mmHg or specific treatment of previously diagnosed hypertension; (5) raised fasting plasma glucose (FPG): ≥6.0 mmol/L or diagnosed type 2 diabetes previously | 1.17(1.01-1.35) |
| Barbosa et al. 2023 (24) | 24-h dietary recall/NOVA food classification: (1) fresh and minimally processed foods;(2) processed culinary ingredients; (3) processed foods, and (4) UPF | MetS was defined by the presence of at least three components according to the Joint Interim Statement—JIS harmonization criteria, with adaptation.  (a)Abdominal obesity: WC≥80cm; (b) Hypertriglyceridemia: TG≥175 mg /dL; (c) Low HDL: HDL<50mg/dL; Hypertension: systolic blood pressure ≥130 or diastolic blood pressure≥85mmHg; (e) Diabetes mellitus:HbA1C ≥6.5mg/dL, or being under drug treatment for hypertriglyceridemia, hypertension or diabetes. | Q4 vs Q1; PR:1.09;  95%CI:0.89~1.32 |
| Canhada et al.2023(25) | FFQ/Monteiro et al. (2016):(1) non-or minimally processed foods and culinary ingredients;(2) processed foods; and (3) UPFs | MetS was defined by the presence of at least three of the five following components:high fasting glucose level (≥100 mg/dL or use of hypoglycemic medication), high triglyceride levels(≥150 mg/dL or use of fibrates and/or nicotinic acid), low HDL cholesterol level(<40 mg/dL for men and <50 mg/dL for women, or use of fibrates and/or nicotinic acid), high blood pressure (systolic blood pressure ≥130 mmHg and/or diastolic blood pressure≥85 mmHg or confirmed use of antihypertensive medication),and abdominal obesity (waist circumference ≥94 cm for men and ≥80 cm for women). | Q4 vs Q1; PR:1.19; 95%CI:1.07-1.32 |
| Tavares et al. 2012(26) | FFQ/Monteiro et al. (2012): unprocessed /minimally processed foods (Group1); processed culinary ingredients (Group2); or ultra-processed ready-to-eat or ready- to-heat food products (Group 3) | MetS classification was based on the definition proposed by De Ferranti et al., adapting the cut-off of hyper-glycaemia. Adolescents were classified as having MetS when three or more of the following components were altered: (i) serum glucose ≥ 100 mg/dl; (ii) systolic blood pressure (SBP) and/or diastolic blood pressure (DBP; mmHg)>90th percentile; (iii) HDL-C <50 mg/dl for girls (12–19 years) and boys (12–14 years) or <45 mg/dl for boys (15–19 years); (iv) TAG ≥ 100 mg/dl; and (v) WC(cm) ≥ 75th percentile. | PR:2.49; 95%CI:1.24~3.57 |
| Martínez Steele  et al.2019(27) | 24h dietary recall/Monteiro et al. (2019): unprocessed or minimally processed foods, processed culinary ingredients, processed foods and ultra-processed foods | Metabolic syndrome was defined as meeting three or more of the following criteria.Elevated waist circumference ≥102 cm in males or ≥ 88 cm in females;Elevated fasting serum triglycerides ≥150 mg/dL (or current prescription treatment for dyslipidemia);Reduced high-density lipoprotein (HDL) cholesterol < 40 mg/dL in men or < 50 mg/dL in women (or current prescription treatment for dyslipidemia);Elevated blood pressure: Systolic blood pressure ≥ 130 mmHg and/or diastolic blood pressure ≥ 85 mmHg(or currently taking antihypertensive medication);Elevated fasting plasma glucose ≥100 mg/dL (or currently taking insulin or an oral hypoglycemic medication) | Q5 vs Q1; PR:1.20; 95%CI:1.07~1.35 |
| Lavigne-Robichaud et al. 2018(28) | 24-h dietary recall/NOVA food classification:unprocessed or minimally processed (Group1); culinary ingredients (Group2);processed products(Group3); ultra-processed food products (Group 4). ultra-processed products include carbonated drinks, salty or sweet  snacks, commercial breads, cereals or energy bars, etc.  cereals, margarine, processed meats, etc. | The presence of MetS among adult Cree was assessed according to latest harmonized definition.of at least three criteria. Central obesity: Waist circumference (South Asian)Men: ≥90 cm,Women: ≥80 cm;TAG ≥1.70 mmol/l; HDL cholesterol: Men: ≤1.03 mmol/lWomen: ≤1.29 mmol/l;  Fasting plasma glucose ≥5.6 mmol/l; Hypertension ≥130/85 mmHg. | Q5 vs Q1;OR:1.90; 95%CI:1.14~3.17 |
| Ivancovsky-Wajcman et al. 2021 (29) | FFQ/NOVA food classification; The UPF groups include several types of sweets,  snack products, industrial bread, cereals, margarine, sauces, processed meat, soft drinks, “energy” drinks, alcoholic beverages, sweetened yogurts (including “fruit” yogurts), spreads, and prepared pizza and pastries. | MetS was defined according to the American Heart Association by the presence of at least three of five criteria; impaired fasting glucose (fasting glucose≥100 mg/dL), hypertension [systolic blood pressur(BP)/diastolic BP ≥130/80 mmHg and/or medication], low levels of high-density lipoprotein cholesterol(HDL)(HDL<40/50 mg/dL among men and women respectively), hyper-triglyceridemia (triglycerides (TGs) ≥150 mg/ dL or medications], and abdominal obesity (waist circumference ≥ 88 cm and 102 cm among women and men respectively). | High vs low; OR:1.88;  95%CI:1.31~2.71 |
| Nasreddine et al. 2018(30) | FFQ/NOVA food classification: minimally processed foods; processed culinary ingredients; processed foods; ultra-processed foods | MetS was diagnosed based on the harmonized definition from the International Diabetes Federation, whereby participants were classified as having the MetS if they had three of the five following cardio-metabolic risk factors: (i) elevated TAG level (≥150 mg/dl);(ii) low HDL-C level (<40 mg/dl for men, <50 mg/dl forwomen); (iii) elevated blood pressure (systolic ≥130 mmHgand/or diastolic ≥85 mm Hg); (iv) elevated fasting glucose level (≥100 mg/dl); and (v) elevated waist circumference(≥94 cm for men, ≥80 cm for women). | Medium/high adherence vs low adherence;OR:1.11;  95%CI:0.26~4.65 |
