# Supplementary figures and images for: Ultra-processed food consumption and increased risk of metabolic syndrome: a systematic review and meta-analysis of observational studies
Source: Front Nutr. 2023 Jun 9;10:1211797. doi: 10.3389/fnut.2023.1211797 (PMC10288143; doi:10.3389/fnut.2023.1211797)

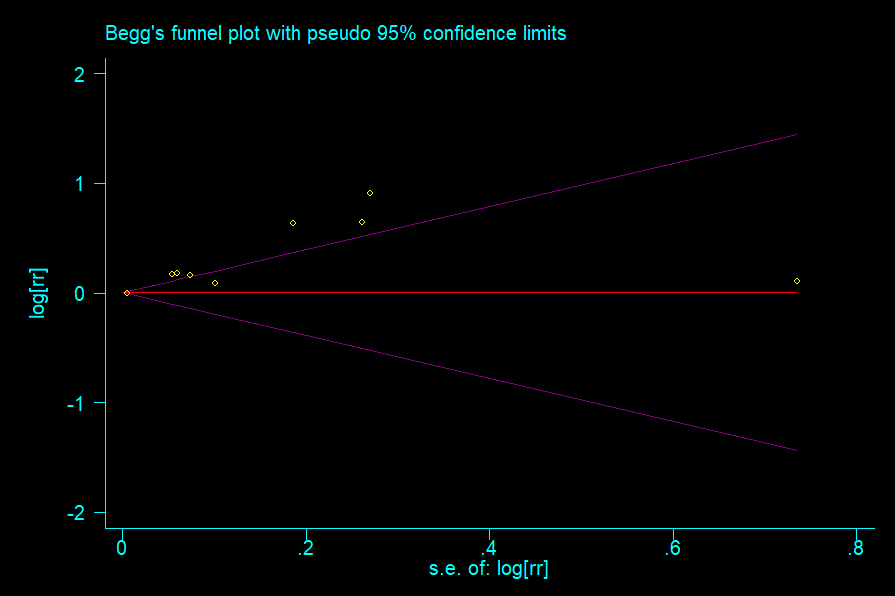

Supplement: Supplementary file 3 [file Image_1.tif]

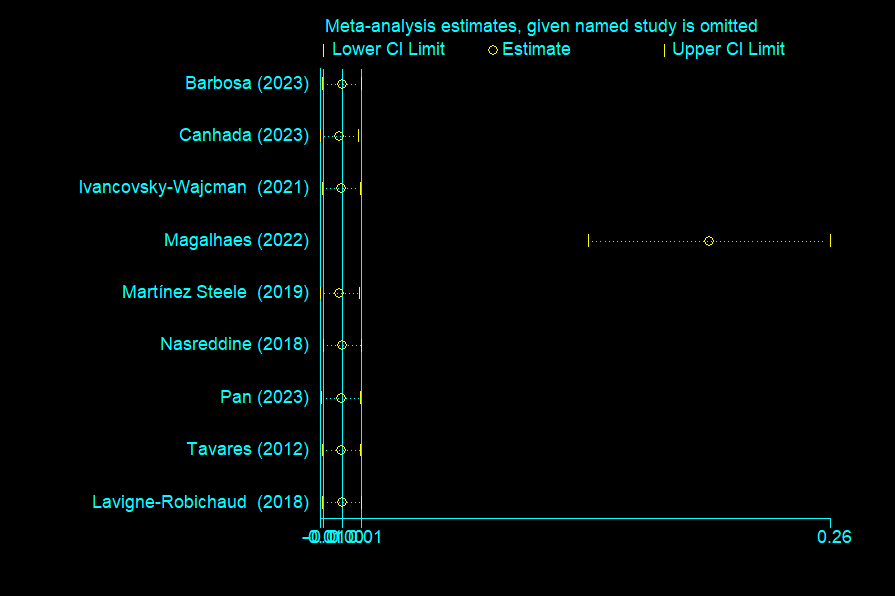

Supplement: Supplementary file 4 [file Image_2.tif]

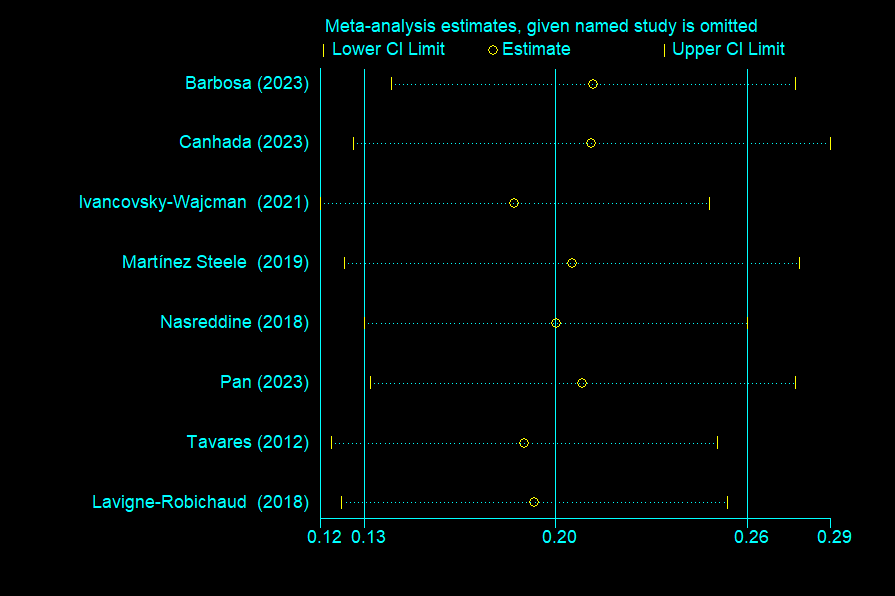

Supplement: Supplementary file 5 [file Image_3.tif]
